# Development of Dual-Arm Human Companion Robots That Can Dance

**DOI:** 10.3390/s24206704

**Published:** 2024-10-18

**Authors:** Joonyoung Kim, Taewoong Kang, Dongwoon Song, Gijae Ahn, Seung-Joon Yi

**Affiliations:** Electrical Engineering Department, Pusan National University, Busan 43241, Republic of Korea; kjykjy98@pusan.ac.kr (J.K.); touy1@pusan.ac.kr (T.K.); dongwoon@pusan.ac.kr (D.S.); dksrlwo@pusan.ac.kr (G.A.)

**Keywords:** dual-arm robot, human–robot interaction, gesture-based interaction, dancing robot

## Abstract

As gestures play an important role in human communication, there have been a number of service robots equipped with a pair of human-like arms for gesture-based human–robot interactions. However, the arms of most human companion robots are limited to slow and simple gestures due to the low maximum velocity of the arm actuators. In this work, we present the JF-2 robot, a mobile home service robot equipped with a pair of torque-controlled anthropomorphic arms. Thanks to the low inertia design of the arm, responsive Quasi-Direct Drive (QDD) actuators, and active compliant control of the joints, the robot can replicate fast human dance motions while being safe in the environment. In addition to the JF-2 robot, we also present the JF-mini robot, a scaled-down, low-cost version of the JF-2 robot mainly targeted for commercial use at kindergarten and childcare facilities. The suggested system is validated by performing three experiments, a safety test, teaching children how to dance along to the music, and bringing a requested item to a human subject.

## 1. Introduction

Current mobile service robots can be categorized into three different types by their arm configurations: basic service robots without arms or manipulators [1,2], mobile manipulation robots with a single arm for manipulating objects [3,4], and human companion robots with two human-like arms mainly used for gestures [5,6,7]. Many human companion robots, whose main focus lies upon interacting with humans, are equipped with two anthropomorphic arms that can be used for gesture-based human–robot interactions. Dancing can be regarded as the most dynamic form of gestures, and there have been a number of approaches to building robots that can dance like humans [8,9,10,11,12]. However, such robots are usually only capable of slow and gentle dance motions, as they cannot replicate highly dynamic dancing motions due to their joint velocity and acceleration limits. These limitations prevent them from replicating the highly dynamic and energetic movements seen in human dancing, which require rapid changes in speed and direction.

**Figure 1:**
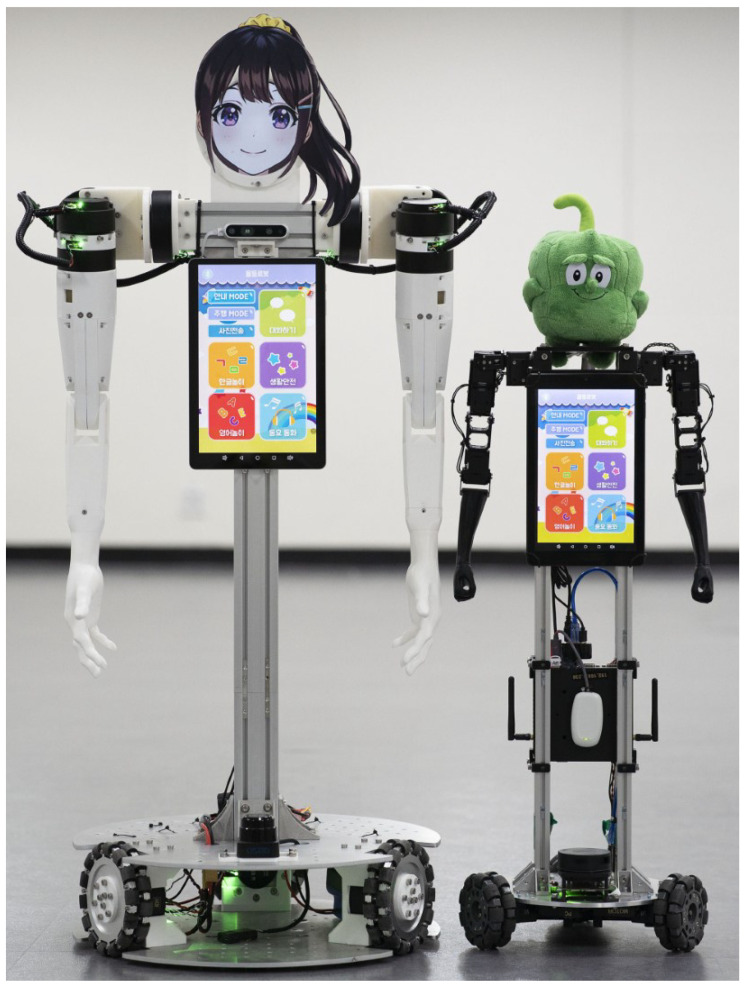
The JF-2 (left) and JF-mini (right) human companion robots without casing.

In this work, we present the JF-2 robot, a mobile home service robot equipped with a pair of anthropomorphic arms actuated by torque-controlled QDD actuators, which use high-torque, high-speed motors with a large stator radius and low gear ratio reducers. Thanks to the low inertia of the arm mechanism and responsive actuators with a low gear reduction ratio, the robot’s arms can replicate dynamic human dance motions, while being compliant to be safe to the people around it. We have also developed the scaled-down, low-cost version of the robot, JF-mini, which is aimed at commercial deployment at kindergartens and childcare centers. Both robots are shown in Figure 1. To validate the performance of the suggested platform, we present a safety test and two service tasks executed by the robots, the dancing along task and the gift delivery task.

The rest of this paper proceeds as follows. Section 2 describes the hardware structure of the two robots. Section 3 presents the software system used in the proposed system. Section 4 presents the experimental results, which include the safety test, the dancing along task, and the gift delivery task. Finally, we conclude with the future direction of this work in Section 5.

## 2. Hardware Architecture

**Figure 2:**
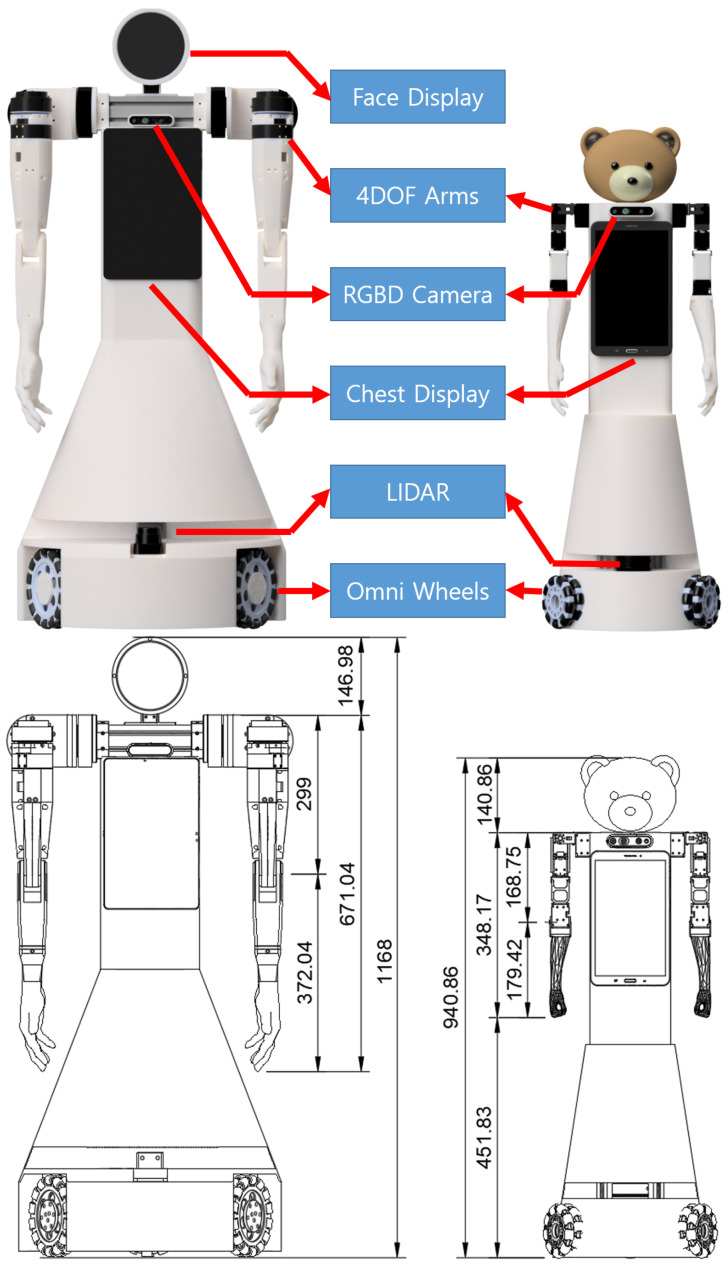
Hardware configurations and dimensions of the JF-2 and JF-mini robots.

We have developed two human companion robots, JF-2 and JF-mini, as shown in Figure 2. JF-2 is a full-sized robot with arm dimensions close to an adult human, which uses fast, torque-controlled QDD actuators to imitate fast human arm movements. JF-mini is a scaled-down, low-cost version of JF-2. In the following subsections, we present each subsystem of the JF-2 and JF-mini hardware.

### 2.1. Arm

**Figure 3:**
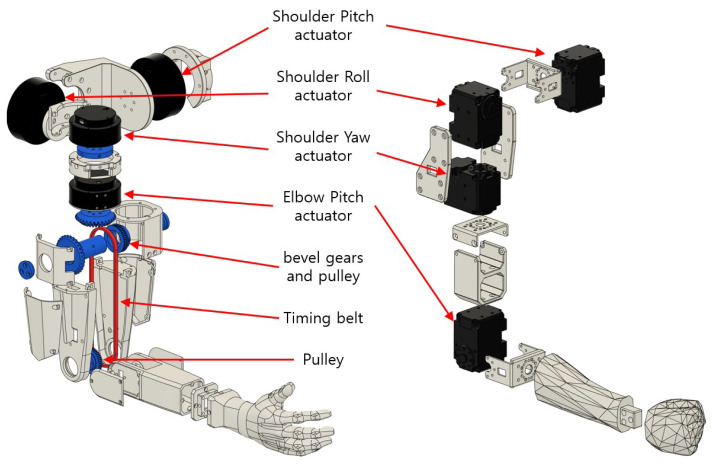
Four-DOF arm mechanisms of the JF-2 and JF-mini robots.

To make the robots capable of replicating arbitrary human arm motions, we have placed two 4-DOF arms on each robot. This is because the human arm, excluding the wrist, can be modeled as a 4-DOF manipulator [13]. Each arm consists of three shoulder joints, which are pitch, roll, and yaw joints in sequence, followed by the elbow pitch joint. The three shoulder joint rotation axes intersect at a single point. Although both robots share the same arm joint sequence and link proportions, the two arms are very different in terms of their mechanism, as shown in Figure 3.

JF-2 uses QDD actuators to drive the joints of its arm, which are highly backdrivable and can react faster to the control signal due to their low gear reduction ratio (9:1 for shoulder pitch and roll actuators, and 6:1 for shoulder yaw and elbow joints). To minimize the arm inertia so that the arm can accelerate as fast as possible, all actuators are mounted together close to the shoulder, and the elbow joint is driven by a timing belt to minimize the arm inertia. Table 1 compares the mass and link inertia properties of the upper arm, lower arm, and hand for the JF2, LIMS, and a human. The upper arm has an inertia of 5795kg·mm², which is 2.5 times lower than a human’s, which is 14,845 kg·mm². The lower arm has an inertia of 320 kg·mm², which is 30 times lower than a human’s, which is 9355 kg·mm². The weight of the JF2 arm moving part is 1.04 kg. Two arms are torque-controlled at 1 kHz using two separate CAN bus channels, where gravity compensation torques and PD control torques are calculated and sent to joints to move the arms to target joint angles. As a result, the arm of the JF-2 robot can move very quickly (up to 1000 degrees per second) while being compliant and safe to touch during movement.

JF-mini has a much simpler arm design, where Dynamixel XL430-W250-T intelligent servomotors (Robotis, Seoul, Republic of Korea) directly actuate each joint. As JF-mini has shorter and much lighter arms compared to JF-2, we have found the Dynamixel actuators to be fully capable of actuating the arm joints according to arbitrary human arm motions.

### 2.2. Mobile Base

The omnidirectional mobility of a robot helps many tasks a service robot may serve, and it is especially helpful for human companion robots, which are required to keep facing a human subject while moving in other directions. For both of our robots, we have chosen a three-omni-wheel setup as the drivetrain as it is the simplest omnidirectional setup and can be cleanly mounted on circular bases.

The diameter of the bases is determined by the shoulder width of the robots, which are 510 mm and 300 mm, respectively, for the JF-2 and JF-mini robots. For a larger JF-2 robot, we use a 152 mm dual-aluminum omni wheels directly driven by RMD-X8 QDD actuators (MyActuator, Suzhou, China). For the JF-mini robot, we use 100mm plastic omni wheels, which are driven by Dynamixel XM430-W210 intelligent actuators. The maximum calculated speed of each platform is 1.4 m/s for JF-2 and 0.28 m/s for JF-mini.

### 2.3. Chest Display and Sensor

**Figure 4:**
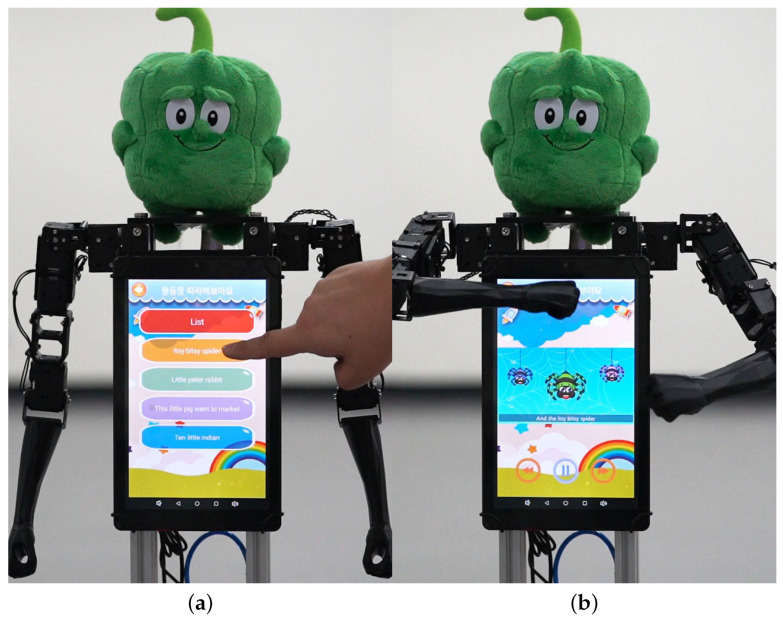
Use cases of the chest display. (**a**) Robot control. (**b**) Synchronously showing an animation while dancing.

Both robots have touch-enabled displays at the chest area, which can be used to either control the robot or provide various multimedia content, as shown in Figure 4. Instead of connecting a dedicated display to the control PC, we use standalone android tablets that run dedicated control app instead. To control the robot, the android tablet accepts the touch inputs and voice inputs from the human operator, and sends the command to the control PC via Bluetooth connection.

In addition to the chest display, two robots are equipped with a RealSense RGBD camera (Intel, Santa Clara, CA, USA) at the chest, which can be used for detecting humans and objects in front of the robot, as well as recording human posture in 3D to imitate human dance motions.

### 2.4. Head

**Figure 5:**
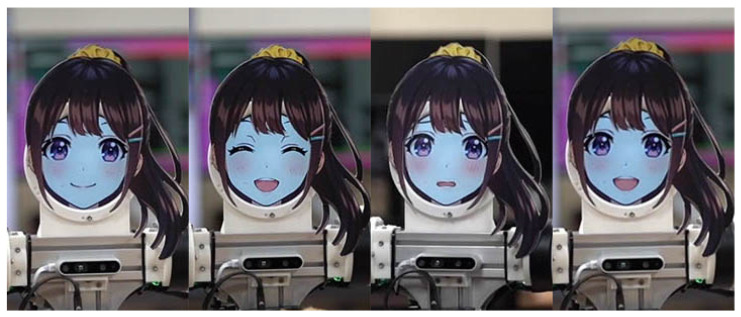
Various facial expressions with the circular LCD head.

Another main difference between the two robots is their head mechanism. For a smaller JF-mini robot, we mounted a stuffed doll at the head location as a non-functional head. However, for the JF-2 robot, we used a head with a 5-inch circular LCD display to show various facial expressions in real time, which are shown in Figure 5. We used separate hair parts, which were magnetically attached over the face and could be easily swapped for different hairstyles. The circular LCD only displayed the facial part of the virtual human, which could be updated in real time to show various expressions. We have found that the resulting setup is natural-looking and aesthetically pleasing in most bright areas and view angles.

### 2.5. Control and Power

Both robots are controlled by a small industrial PC, which is located at the center column of the robot and is connected to base LIDAR and XBOX 360 controller (Microsoft, Redmond, WA, USA) wireless adapter. For the JF-2 robot, the control PC is connected to three separate CAN bus adapters via a USB, which control two arms and base actuator chains separately. In addition, the head display is connected to the control PC’s HDMI output. A Kobalt 24 V drill battery pack (Kobalt, Union City, NJ, USA) is used to drive power-hungry QDD actuators, and a separate USB-PD battery pack is used to power the control PC. For the JF-mini robot, all Dynamixel intelligent actuators are connected in a single bus and controlled by an RS-485 interface, and a single 12 V battery pack powers both the control PC and the actuators.

## 3. Software Architecture

**Figure 6:**
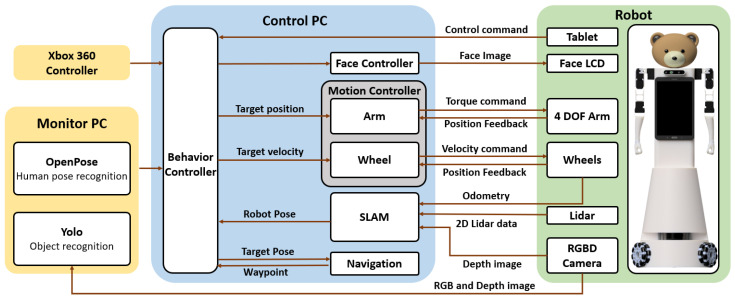
Software architecture of the JF-2 and JF-mini robots.

The software framework we use for two robots has its roots in the RoboCup international robotic soccer competition [16]. It is designed to be highly modular to support a variety of robotic hardware and be quickly ported on new robot platforms with minimal effort, as well as supporting various robotic simulators. The software framework was mainly used for mobile home service robots, which is summarized in [17]. Figure 6 shows the overall structure of the current software framework’s JF-2 and JF-mini robots’ use.

### 3.1. SLAM and Navigation

To build the map of an environment the robot needs to operate in, we first control the robot manually using the XBOX 360 controller (Microsoft, Redmond, WA, USA), while running the ROS hector_slam package [18]. While building the map of the environment, the operator also marks a number of waypoints where the robot is supposed to visit during various service tasks, such as the item pickup position, item release position, human interaction position, and so on. Once the map building is completed, the robot uses the ROS amcl package [19], which uses a particle filter, a pre-built 2D map of the environment, and 2D laser scan data to localize the robot. To navigate the robot to the target waypoint, we use a custom motion planner using the A* algorithm and visibility graph to efficiently traverse a mixed indoor environment, which contains both narrow corridors and open spaces. The suggested system has obstacle avoidance algorithms in multiple layers and has both soft and hard emergency stop systems. Our software was successfully validated in the RoboCup@Home 2020 and World Robot Summit 2020 Partner Robot Challenge (WRS 2020 PRC) league, which led us to obtain first place in both competitions [20].

### 3.2. Object and Human Detection

We use a single RGBD camera as the main sensor for detecting humans and known objects in front of the robot. Known objects are first detected from RGB images by utilizing the YOLO real-time object detection algorithm, and the detected object class and object bounding box are used with corresponding depth images to determine the 3D position and orientation of the object. To detect humans from RGBD data, we use the OpenPose [21] human keypoint algorithm to detect humans in the RGB image, and then we use the depth image to calculate the 3D positions of human keypoints. As the OpenPose algorithm alone may return false positives in complex scenes, we check the resulting 3D limb lengths to filter out too small or too large human candidates.

### 3.3. Human Motion Acquisition

**Figure 7:**
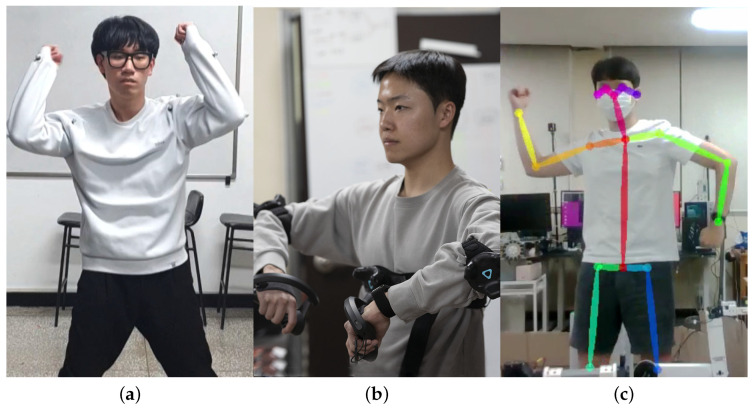
Three different human motion capturing methods. (**a**) Motion capture-based. (**b**) VR tracker-based. (**c**) Keypoint detection-based.

We used three different methods to capture human motion data for dancing, which are shown in Figure 7. We first use the OptiTrack motion capture system to generate the reference human motion data, which have a 240 Hz refresh rate and tracking accuracy of 0.3 mm. Although this motion capture setup provides very high-quality motion data, it requires an expensive hardware setup that cannot be transported easily.

The second capturing method utilizes commercial virtual reality (VR) controllers and trackers. We use 4 SteamVR 2.0 base stations (HTC, Taipei, Taiwan) to minimize occlusion issues, and we use 3 trackers and 2 controllers to record the chest, elbow, and hand poses of the operator. Although they have lower positional accuracy compared to motion capture devices, they still have sub-millimeter tracking precision [22]. We have found them perfectly suitable for human motion recording.

Finally, we use the onboard RGBD camera and the OpenPose [21] human keypoint detection algorithm to determine 3D human posture. Human keypoints are first detected from the RGB image, and 3D keypoint positions are calculated by matching the depth image. This method has the advantage of not requiring any additional sensors or trackers; however, it needs special care to disambiguate occlusion cases.

In conclusion, we decided to utilize virtual reality controllers and trackers for the following reasons. Firstly, this method significantly simplifies the setup process compared to the OptiTrack system, making it much easier to configure the environment for motion capture. Secondly, despite the higher precision of the OptiTrack system, the difference in the robot’s actual motion between methods 1 and 2 is subtle, as the motion data must still be adapted to the robot’s hardware, reducing the benefit of the increased accuracy. Lastly, the third method using the onboard RGBD camera and OpenPose suffers from occlusion issues inherent in RGB images. Occlusions in one dimension can lead to inaccurate or incomplete motion capture data, making this method unreliable in certain scenarios. Therefore, VR controllers and trackers provide a more practical and equally effective solution for our needs.

**Figure 8:**
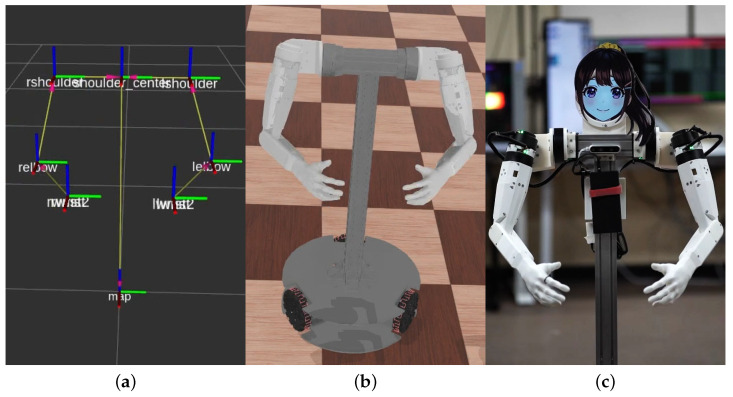
Human motion-replicating process. (**a**) Shoulder, elbow, and hand 3D positions assuming zero chest tilt and roll angles. (**b**) Retargeted joint angles in a simulated environment. (**c**) Arm postures realized with JF-2 robot.

Once the 3D keypoint positions are acquired, arm joint angles are calculated assuming zero chest tilt and roll angles, and they are retargeted to robot arms, as shown in Figure 8.

### 3.4. Behavior Control

We use a number of finite-state machines (FSMs) to govern various behavior of the robot. Body FSMs handle the movement of the mobile base, which includes navigating to the target pose, approaching to the manipulation target or human subject within a predetermined distance, and freely moving around based on the controller input. Arm FSMs handle the movement of two anthropomorphic arms, which includes playing back one of the dance motions recorded previously, picking up an object, and giving an object to a human subject. Finally, Task FSMs govern the high-level behavior of the robot according to input provided by the chest touch display, as well as sensory inputs such as LIDAR scan data and perception results. For example, the kindergarten stroll task includes navigating through predefined waypoints, greeting people at the waypoints and waiting for the command, and dancing along with the video being played at the chest display if asked.

## 4. Experimental Results

The JF-2 and JF-mini robots are well equipped for a large number of service tasks, as they can navigate, detect humans and objects in 3D, and interact with humans using the chest touch display, face expressions, and arm motions. In this section, we present the results of the safety test and two tasks we experimented with.

### 4.1. Safety Test

**Figure 9:**
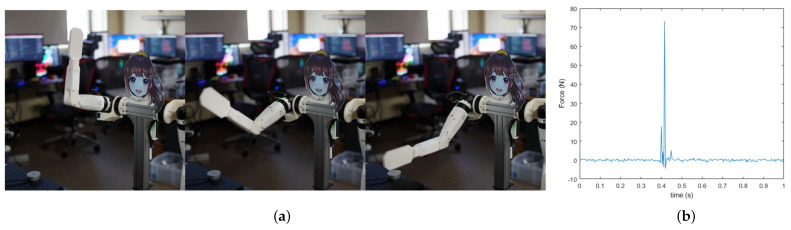
(**a**) Snapshots of the safety test. (**b**) Time-force results.

We conducted high-speed motion impact tests using the entire arm to verify the safety of the possible robot collision. We measured the force repeatedly by the dummy hand directly striking a force/torque sensor (RFT60-HA01 (Robotous, Seongnam, Republic of Korea)) fixed on the desk, as shown in Figure 9a. To maximize the impact force, the robot arm strikes with a downward motion with gravity without any shock-absorbing materials used. Figure 9b shows the force measurements over time, with the contact occurring in one second. In 20 attempts, the average peak force, excluding outliers, was measured at 94.6 N. Even though the experiment was conducted in a challenging setup, the results demonstrate that the system’s maximum possible impact is lower than the human force pain thresholds [23].

### 4.2. Dancing Along Task

These tasks assume a kindergarten environment, where the robot freely roams inside a kindergarten and teaches children how to dance along to the music by providing a live demonstration. We first selected four children’s songs, and we updated the dedicated control app for the chest tablet so that the human operator could select one of the songs to be played on the tablet. Then, we recorded the dance motions of the songs using the VR tracker method. When a song is selected by the chest display, a dancing start signal is sent to the control PC so that the robot can play back the selected dance motion in sync with the video playback.

**Figure 10:**
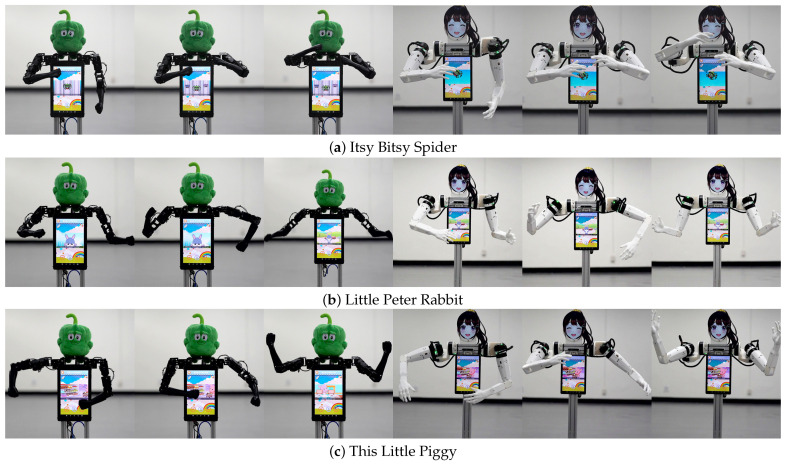

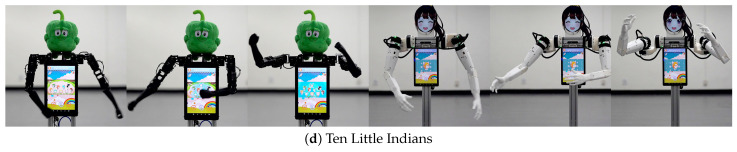
JF-2 and JF-mini robots dancing along to four different children’s songs.

Figure 10 shows two robots dancing along to the four children’s song we selected. We found that the JF-mini robot, even with its lower joint velocity limit, can still play back dancing motions for children’s songs without noticeable lag (video available at https://youtu.be/2tMR-mIfsY4, accessed on 4 July 2024).

### 4.3. Gift Delivery Task

**Figure 11:**
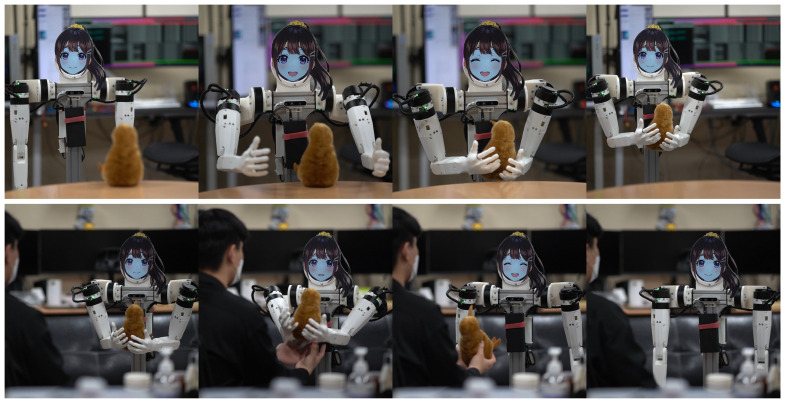
JF-2 robot executing the gift delivery task.

The second task we tested was an extension of one of the common service robot tasks. In this task, the robot was asked to bring an object to the human operator. Then, the robot navigated to the item pickup position, found the objects using the 3D object perception, and picked up the object using both hands. Once the robot picked up the object, the robot navigated back to the operator and handed over the object. Although the anthropomorphic arms of the JF-2 robot are not designed primarily for object pickup, we have found that the robot can pick up a wide range of objects thanks to the compliance of the joints. In addition, compared to regular home service robots, the expressive head of the JF-2 provides additional personality traits to the robot. The robot’s LCD display can show a variety of facial expressions, enhancing the interaction experience. These varied facial expressions make the robot appear more engaging and relatable to users. Figure 11 shows the JF-2 robot executing the gift delivery task.

## 5. Conclusions

**Figure 12:**
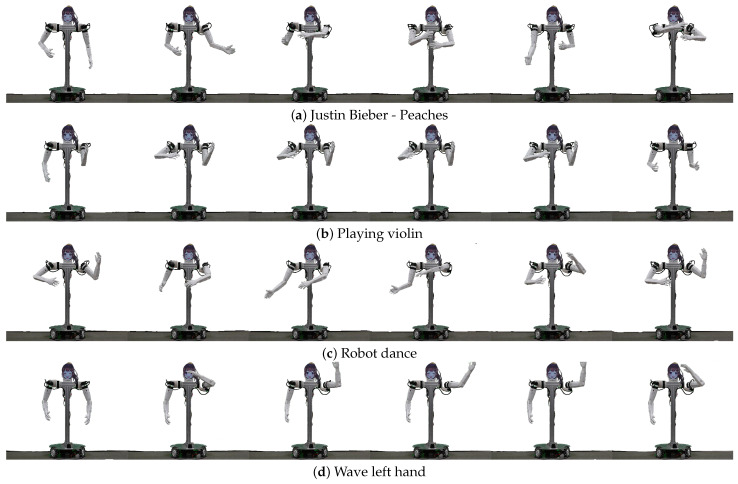
Examples of dance motion by JF2.

We developed two human companion robots with anthropomorphic arms, JF-2 and its scaled-down version JF-mini. In addition to the common capabilities of indoor service robots, which include perception, navigation, and verbal and nonverbal human–robot interactions, JF-2 can replicate fast human arm motions utilizing its low-inertia, torque-controlled, high-speed arms. In addition, JF-2 is equipped with an expressive head with a circular LCD display. We validated the safety of JF-2 through impact force measurement experiments. JF-2 was tested for the gift delivery task, wherein the robot fetches a requested item and delivers it to the human operator while utilizing facial expressions for communication. Also, both robots successfully completed the dancing along task, where the robots taught children how to dance along to the music through live demonstrations (Figure 12). We expect to enlarge the use cases of these robots to a wider range of HRI tasks, such as serving at a party, escorting a person, and being a receptionist at a desk.

## Figures and Tables

**Table 1:** Mass/link inertia comparison.

	Mass (kg)			Link Inertia (kg·mm^2^)		
	Upper Arm	Lower Arm	Hand	Upper Arm	Lower Arm	Hand
JF2	0.75	0.11	0.18	2,781	320	603
LIMS [14]	1.28	0.64	0.32	18,796	6895	694
Human [15]	2.5	1.45	0.53	14,845	9355	1370

## Data Availability

Dataset available on request from the authors.
